# Structural insights for neutralization of Omicron variants BA.1, BA.2, BA.4, and BA.5 by a broadly neutralizing SARS-CoV-2 antibody

**DOI:** 10.1126/sciadv.add2032

**Published:** 2022-10-05

**Authors:** Sanjeev Kumar, Anamika Patel, Lilin Lai, Chennareddy Chakravarthy, Rajesh Valanparambil, Elluri Seetharami Reddy, Kamalvishnu Gottimukkala, Meredith E. Davis-Gardner, Venkata Viswanadh Edara, Susanne Linderman, Kaustuv Nayak, Kritika Dixit, Pragati Sharma, Prashant Bajpai, Vanshika Singh, Filipp Frank, Narayanaiah Cheedarla, Hans P. Verkerke, Andrew S. Neish, John D. Roback, Grace Mantus, Pawan Kumar Goel, Manju Rahi, Carl W. Davis, Jens Wrammert, Sucheta Godbole, Amy R. Henry, Daniel C. Douek, Mehul S. Suthar, Rafi Ahmed, Eric Ortlund, Amit Sharma, Kaja Murali-Krishna, Anmol Chandele

**Affiliations:** ^1^ ICGEB-Emory Vaccine Center, International Center for Genetic Engineering and Biotechnology, New Delhi-110 067, India.; ^2^ Department of Biochemistry, Emory University School of Medicine, Atlanta, GA 30322, USA.; ^3^ Department of Pediatrics, Emory University School of Medicine, Emory University Atlanta, GA 30322, USA.; ^4^ Department of Microbiology and Immunology, Emory University School of Medicine, Emory University, Atlanta, GA 30322, USA.; ^5^ Emory Vaccine Center, Emory University, Atlanta, GA 30322, USA.; ^6^ Kusuma School of Biological Sciences, Indian Institute of Technology, New Delhi-110 016, India.; ^7^ Department of Pathology and Laboratory Medicine, Emory University School of Medicine, Atlanta, GA 30322, USA.; ^8^ Department of Pathology, Brigham and Women’s Hospital, Boston, MA 02215, USA.; ^9^ Shaheed Hasan Khan Mewat Government Medical College, Haryana, India.; ^10^ Division of Epidemiology and Communicable Diseases, Indian Council of Medical Research, New Delhi-110 029, India.; ^11^ Vaccine Research Center, National Institute of Allergy and Infectious Diseases, National Institutes of Health, Bethesda, MD 20892, USA.; ^12^ ICMR-National Institute of Malaria Research, Dwarka, New Delhi-110 077, India.; ^13^ Structural Parasitology Group, International Center for Genetic Engineering and Biotechnology, New Delhi-110 067, India.

## Abstract

In this study, by characterizing several human monoclonal antibodies (mAbs) isolated from single B cells of the COVID-19–recovered individuals in India who experienced ancestral Wuhan strain (WA.1) of SARS-CoV-2 during early stages of the pandemic, we found a receptor binding domain (RBD)–specific mAb 002-S21F2 that has rare gene usage and potently neutralized live viral isolates of SARS-CoV-2 variants including Alpha, Beta, Gamma, Delta, and Omicron sublineages (BA.1, BA.2, BA.2.12.1, BA.4, and BA.5) with IC_50_ ranging from 0.02 to 0.13 μg/ml. Structural studies of 002-S21F2 in complex with spike trimers of Omicron and WA.1 showed that it targets a conformationally conserved epitope on the outer face of RBD (class 3 surface) outside the ACE2-binding motif, thereby providing a mechanistic insights for its broad neutralization activity. The discovery of 002-S21F2 and the broadly neutralizing epitope it targets have timely implications for developing a broad range of therapeutic and vaccine interventions against SARS-CoV-2 variants including Omicron sublineages.

## Teaser

A rare human monoclonal antibody neutralizes all SARS-CoV-2 Omicron variants by targeting a conserved epitope on the RBD.

## INTRODUCTION

The ongoing coronavirus disease 2019 (COVID-19) pandemic caused by severe acute respiratory syndrome coronavirus 2 (SARS-CoV-2) has resulted in roughly 552 million cases and 6 million deaths worldwide (*1*). Intense global efforts are being pursued to develop, evaluate, and implement vaccines or other medical countermeasures, including monoclonal antibody (mAb) therapy (*2*, *3*). Widespread transmission and key mutations have led to the emergence of viral variants that escape neutralization by therapeutic antibodies as well as natural and vaccine-acquired immunity (*4*–*8*). Most therapeutic mAbs currently licensed for use against SARS-CoV-2 have shown reduced neutralizing activity against the Omicron (B.1.1.529) variant and its sublineages (*7*–*10*). This highlights a continuous need to identify mAbs that are effective against emerging variants.

Like other human coronaviruses, the spike protein of SARS-CoV-2 facilitates the entry of virus into host cells and comprises two subunits, S1 and S2 (*11*). The receptor binding motif (RBM), a region of the receptor binding domain (RBD) present in the S1 subunit, interacts with the host cell receptor angiotensin-converting enzyme 2 (ACE2), whereas the S2 subunit is involved in the fusion of the viral and host cell membranes (*11*). The RBD is the major antigenic site in spike and, based on their epitopes, two classification schemes have been proposed to divide RBD-specific mAbs into either four (classes 1 to 4) or seven (RBD1 to RBD7) categories (*2*, *12*, *13*). An unprecedented number of mutations (>10) in the RBM of Omicron and its sublineages contribute to resistance to currently available therapeutic mAbs (*6*, *7*, *9*, *14*).

Here, we identified a broad SARS-CoV-2 neutralizing antibody 002-S21F2 that potently neutralizes most SARS-CoV-2 variants, including all Omicron sublineages (BA.1, BA.2, BA.2.12.1, BA.4, and BA.5). The cryo–electron microscopy (cryo-EM) structure of the spike-002-S21F2 complex reveals binding to an infrequently mutated class 3 epitope on the outer RBD surface. Epitope mutations in Omicron subvariants are accommodated by fortuitous compensatory molecular interactions explaining the remarkably broad neutralization demonstrated by 002-S21F2 antibody.

## RESULTS

### Isolation and characterization of SARS-CoV-2 RBD-specific human mAbs

**Fig. 1.**
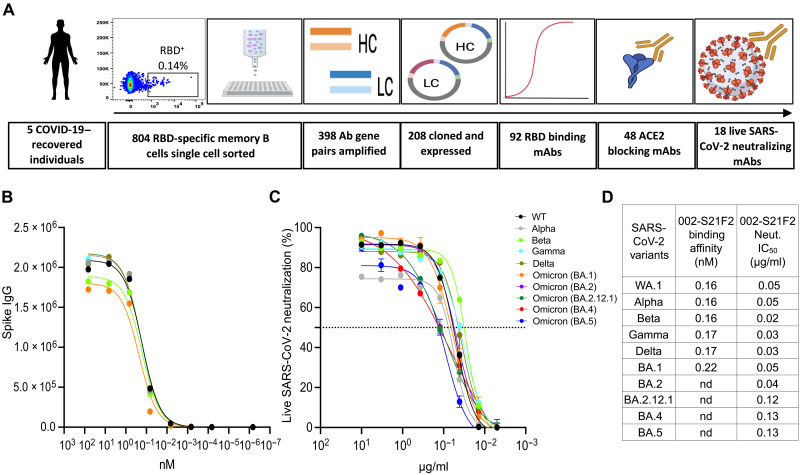
Identification of a broad and potent SARS-CoV-2 RBD-specific human mAb 002-S21F2. (**A**) Overall strategy for the isolation of RBD-specific mAbs described in this study. (**B**) 002-S21F2 was tested for binding to the spike proteins of SARS-CoV-2 WA.1, Alpha, Beta, Gamma, Delta, and Omicron (BA.1) variants of concern (VOCs). (**C**) Authentic live virus neutralization curves of 002-S21F2 for WA.1, Alpha, Beta, Gamma, Delta, and Omicron (BA.1, BA.2, BA.2.12.1, BA.4, and BA.5) SARS-CoV-2 VOCs. Neutralization was determined on Vero-TMPRSS2 cells using a focus reduction assay. (**D**) 002-S21F2–mediated neutralization IC_50_ values were obtained from live SARS-CoV-2 VOC neutralization assays. Affinity constant (*K*_D_) values calculated from the binding curves for two mAbs as measured by the MSD binding assays are plotted. Here, nd stands for not determined.

We previously evaluated the humoral immune responses in 42 COVID-19–recovered individuals who had experienced mild symptoms after the ancestral Wuhan strain (WA.1) transmission in the year 2020 (*15*). We selected five individuals (table S1) who had high SARS-CoV-2 RBD binding titers, high neutralization titers to live SARS-CoV-2 WA.1, and had detectable frequencies of RBD-specific memory B cells (fig. S1, A to C) for the generation of SARS-CoV-2 RBD-specific mAbs. In total, we sorted 804 SARS-CoV-2 RBD fluorescent probe binding class-switched B cells, amplified 398 (~50%) paired heavy- (HC) and light-chain (LC) antibody gene sequences, and successfully cloned and expressed 208 antibodies (Fig. 1A). RBD-based enzyme-linked immunosorbent assay (ELISA) screening resulted in the identification of 92 SARS-CoV-2–specific mAbs (fig. S1D). These mAbs showed an average CDR3 (complementarity-determining region 3) length of 16.3 amino acids, which is typical of a human immunoglobulin G (IgG) repertoire (fig. S1E) (*16*), as well as enriched usage of HC and LC variable region genes belonging to the IGHV3, IGKV1, and IGLV1 families (fig. S1F). Most of these mAbs had a low frequency of somatic hypermutations (SHM) in both their HC and LC, suggesting that they were recently recruited from a naive B cell pool which is typical of a primary infection (fig. S1G). Of these mAbs, 48 blocked the ACE2-RBD interaction (fig. S2A), and 18 (37.5%) successfully neutralized live virus with 50% inhibitory concentration (IC_50_) values ranging from 0.05 to 17 μg/ml (fig. S2B). Antibody 002-S21F2 was the most potent among all the mAbs that neutralized live SARS-CoV-2 WA.1 and thus was selected for comprehensive downstream characterization.

### Antibody 002-S21F2 neutralizes SARS-CoV-2 variants including Omicron and its sublineages

Binding analysis assessed by an electrochemiluminescence multiplex assay [meso scale discovery (MSD)] revealed that 002-S21F2 bound with similar affinities to all tested SARS-CoV-2 variant spike proteins including WA.1, Alpha (B.1.1.7), Beta (B.1.351), Gamma (P.1), Delta (B.1.617.2), and Omicron (B.1.1.529) BA.1 (Fig. 1, B and D). Furthermore, 002-S21F2 bound with picomolar affinity to the prefusion-stabilized WA.1 spike (spike-6p) by biolayer interferometry (BLI) (fig. S3, A and B). Encouraged by these binding results, we tested the live virus neutralization potential of 002-S21F2 against SARS-CoV-2 variants. Antibody 002-S21F2 was capable of broadly neutralizing the Alpha, Beta, Gamma, Delta, and all Omicron subvariants BA.1, BA.2, BA.2.12.1, BA.4, and BA.5 with IC_50_ values of 0.05, 0.02, 0.03, 0.03, 0.05, 0.04, 0.12, 0.13, and 0.13 μg/ml, respectively (Fig. 1, C and D).

### 002-S21F2 is a broadly neutralizing class 3/RBD-5 epitope targeting antibody

**Fig. 2.**
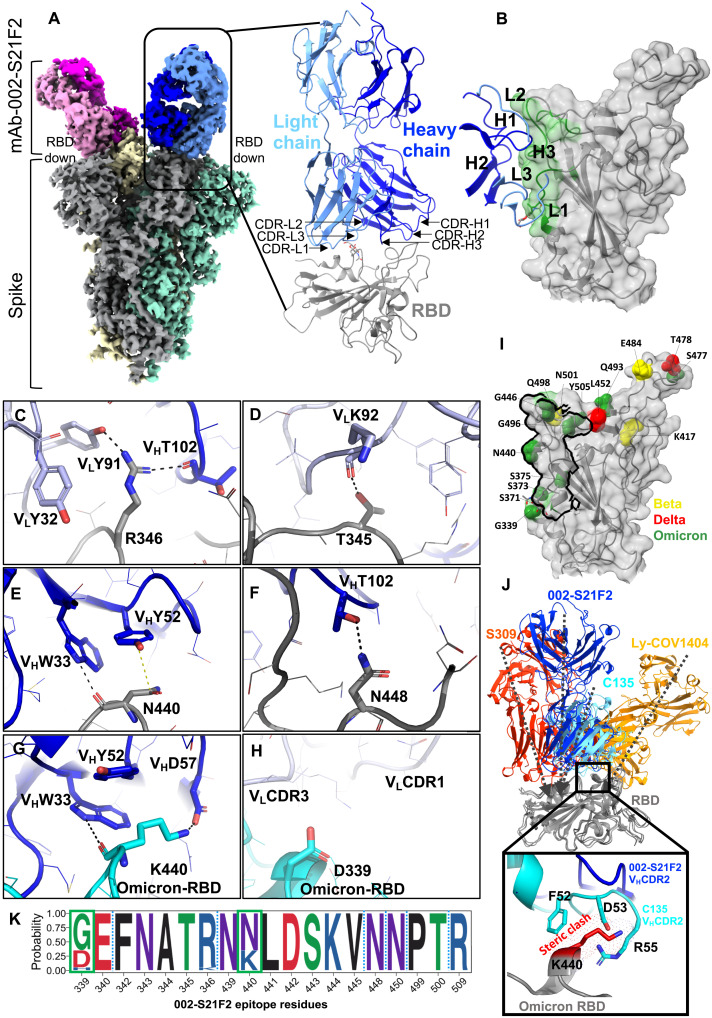
Cryo-EM structure of 002-S21F2 in complex with spike trimer illustrates its binding and neutralization of VOCs. (**A**) Cryo-EM structure of WA.1 spike-6P trimer in complex with mAb 002-S21F2. Overall density map at the contour level of 3.7 𝜎 showing the antibody binding in all RBD down conformation. Each protomer of spike protein is shown in gray, yellow, and green; LC and HC of each Fab region are shown in blue/magenta and light blue/pink, respectively. The model for one of the Fab and RBD is shown in right, and the positions of all CDR regions are labeled. (**B**) Surface representation of RBD with relative positions of all CDR loops. (**C** to **H**) Interaction details at 002-S21F2 and RBD binding interface, WA.1 (C to F) and Omicron (G and H). All hydrogen bonds are shown as black dashed lines and hydrophobic interaction as yellow dashed line. (**I**) locations of Beta (yellow), Delta (red), and Omicron (green) mutations on RBD relative to 002-S21F2 epitope site that is shown as black outline. (**J**) Structural comparison of 002-S21F2 binding mode with other class 3 mAbs, S309, C135, and LY-COV1404; arrows represent their angel of approach on RBD. Zoomed-in view showing the steric clash of Omicron K440 mutation with CDR2 residues in mAb C135. (**K**) Sequence logo representing the sequence conservation of 002-S21F2 epitope based on the sequence alignment of RBD in SARS-CoV-2 variants shown in fig. S9. Residue positions mutated in Omicron subvariants within 002-S21F2 epitope are boxed in green.

To define the molecular features conferring epitope recognition and to understand the mechanism of the broad neutralization spectrum of 002-S21F2 against SARS-CoV-2 variants, we determined the cryo-EM structures of 002-S21F2 full-length IgG in complex with WA.1 and Omicron spike-6P at 3.7 and 4.1 Å, respectively (Fig. 2 and figs. S4 and S5). The cryo-EM structure showed that 002-S21F2 binds to the outer face of the RBD that is accessible in both “down” and “up” conformations and is outside the ACE2-binding motif (Fig. 2, A and B). The interaction buried a total surface area of ~737 Å^2^ with HC and LC contributing ~60 and ~40% of the total interaction, respectively (Fig. 2B). Most of the interactions are mediated through the HC and LC CDR3 regions, and the epitope aligns with RBD-5/class 3 antibodies (*12*, *13*). RBD residue R346 is the main contact point and is sandwiched between the HC CDR3 and LC CDR1 and CDR3 regions. Specifically, the guanidine group of R346 engages in multiple hydrogen bonds involving T102 and Y91 from the HC and LC CDR3, respectively (Fig. 2C) and has the potential for a cation-π stacking interaction involving Y32 from LC CDR1. The T102 hydroxyl in the CDR3 HC also hydrogen bonds with the RBD N448 side chain (Fig. 2F). The other major interaction site involves RBD residue N440, which engages in a direct hydrogen bond with W33 from CDR1 and aligns parallel to the side chain of Y52 in the CDR2 to make CH-π hydrophobic interaction in the HC (Fig. 2E). In addition, the side chain of T345 in the RBD hydrogen bonds with the main chain carbonyl of K92 in the LC CDR3 (Fig. 2D). Most variants of concern (VOCs), with the exception of Omicron, do not contain any mutations within the 002-S21F2 epitope, explaining its broad neutralization ability (Figs. 1C and 2I). All Omicron subvariants (BA.1 and BA.2, BA.2.12.1, BA.4, and BA.5) contain glycine-339 to aspartic acid (G339D) and asparagine-440 to lysine (N440K) mutations within the 002-S21F2 epitope. However, the Omicron spike 002-S21F2 structure reveals identical binding compared to WA.1, and the two structures align with overall Cα backbone root mean square deviation of 0.975 and 0.875 Å in the RBD Fab region (fig. S7). All 002-S21F2 interactions observed in WA.1 remain conserved in Omicron. Furthermore, the side chain of K440 in Omicron RBD makes an additional hydrogen bond with D57 in the HC CDR2 (Fig. 2G), explaining why this change has minimal impact on affinity and neutralization (Fig. 1, B to D). In addition, the G339D mutation in Omicron does not generate any obvious adverse effects in the structure (Fig. 2H).

The antigenic residues targeted by 002-S21F2 broadly neutralizing antibody (bnAb) are highly conserved among current and previous SARS-CoV-2 VOCs (Fig. 2K and figs. S8 and S9). Our structural data agree with neutralization results showing that 002-S21F2 continues to maintain potent neutralization against Omicron variants BA.1, BA.2, BA.2.12.1, BA.4, and BA.5 which harbor epitope mutations G339D and N440K (Figs. 1, C and D, and 2G and figs. S7 and S8). Structural comparison of the 002-S21F2 epitope with other class 3 mAbs, including the two available therapeutic mAbs effective against Omicron—LY-CoV1404 (bebtelovimab) and S309 (sotrovimab)—shows some similarities between the 002-S21F2 and C135 binding sites (Fig. 2J) (*17*, *18*). However, C135 is unable to neutralize Omicron as a lysine mutation at RBD site N440 position would sterically clash with the C135 HC CDR2 (Fig. 2J) (*19*). In support of this, RBD deep mutational scanning shows that an N440K mutation (present in Omicron sublineages) disrupts the RBD-C135 interaction (*20*).

Although 002-S21F2 recognizes an epitope outside the ACE2-binding motif, it may directly block ACE2 interaction through head-to-head interspike cross-linking as observed at saturating spike to IgG concentration (fig. S4C). This corroborates a recent report that positively correlates high neutralization potency to interspike cross-linking ability within the RBD-5/class 3 antibodies (*12*). We also observed a higher ratio of all RBD down conformations (~54% particles) in antibody-bound spike data compared to apo spike-6P (which only shows ~35% of all RBD down conformation particles). Both putative mechanisms may interfere with the ACE2 binding and contribute to neutralization.

### 002-S21F2 bnAb exhibits rare immunogenetic features

**Fig. 3.**
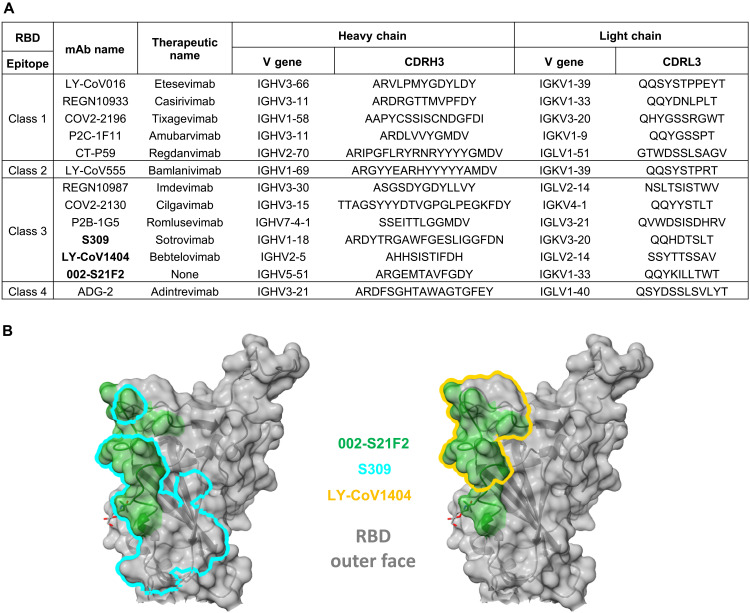
Antibody 002-S21F2 exhibits distinct genetic and epitope contact features in comparison to SARS-CoV-2 therapeutic antibodies. (**A**) Comparison of 002-S21F2 mAb genetic feature with therapeutic mAbs in clinics. Omicron neutralizing mAbs are highlighted in bold. (**B**) Comparison of 002-S21F2 (green) epitope site with S309 (sotrovimab) (cyan outline) and LY-CoV1404 (bebtelovimab) (yellow outline) epitopes on SARS-CoV-2 RBD.

Sequence analysis of 002-S21F2 revealed that its HC variable region is comprised from VH5-51, DH5-24, and JH4 genes; the LC gene uses VK1-33 and JK2 (fig. S6A). Alignment of the 002-S21F2 mAb sequence to its germline sequence revealed four amino acid mutations in the HC that spanned the FR1 and CDR1 regions and three amino acid mutations present in the FR3 and CDR3 regions of the LC (fig. S6B). This low frequency of SHM, 2.7% in the HC and 1.7% in the LC, suggests that the memory B cell that expressed this mAb had not yet undergone extensive selection in the germinal center. Of the 5252 SARS-CoV-2 mAb sequences banked in the CoV-AbDab database (*21*), only two others use this combination of VH5-51 and VK1-33 (fig. S6C). Further comparison of 002-S21F2 with SARS-CoV-2 therapeutic mAbs approved for clinical use revealed no obvious genetic similarities, suggesting that 002-S21F2 exhibits unique genetic characteristics (Fig. 3A).

## DISCUSSION

Structural studies have reported that SARS-CoV-2 bnAbs target only a few antigenic sites on the RBD, which are majorly recognized by class 3 and class 4 mAbs (*12*, *13*, *18*, *19*). Omicron and its sublineages can evade natural and vaccine-generated immunity and pose a threat to immune-compromised, vaccine-hesitant, and unvaccinated adults and children. However, only two of the currently approved therapeutic antibodies have shown neutralization potential to Omicron—an S309 derivative (sotrovimab) and LY-CoV1404 (bebtelovimab). We show that 002-S21F2 potently neutralizes Omicron sublineages BA.1, BA.2, BA.2.12.1, BA.4, and BA.5 and previous VOCs without sacrificing potency. This potent functionality is similar to some recently reported bnAbs bebtelovimab (*17*), S2X324 (*22*), S2K146 (*23*), SA58 (*24*), and 87G7 (*25*) compared to sotrovimab that neutralized BA.1 with ~3-fold higher potency (than WA.1) yet poorly neutralizes BA.2, BA.2.12.1, BA.4, and BA.5 (*9*, *24*). mAb 002-S21F2 is similar to other SARS-CoV-2 RBD-specific bnAbs (LY-CoV1404 and sotrovimab) with respect to all three are class 3 antibodies that recognize overlapping but distinct epitopes on the outer face of RBD (Fig. 3B), compared to 87G7 and S2K146 that recognize a class 1 epitope in the RBM region (*23*, *25*). This suggests that the epitopes defined by the SARS-CoV-2 class 3 bnAbs target distinct antigenic residues on the outer face of the RBD and that this surface may potentially form the basis for an effective vaccine. For example, selective steering of B cell immune responses to the RBD class 3 antigenic sites, defined by 002-S21F2 and bebtelovimab bnAbs, may induce potent antibody responses against SARS-CoV-2 VOC. A similar successful strategy of epitope-focused vaccine candidates has been previously used to guide induction of HIV-1 bnAbs VRC01 and PGT121 that are currently in clinical trials (*26*–*28*). In addition, 002-S21F2 maintains potent neutralization to Omicron variants despite being isolated from a convalescent individual infected in the early months of the pandemic, when only the ancestral SARS-CoV-2 WA.1 strain was reported. In addition, the structural analysis shows that the limited number of SHMs observed in this near germline bnAb 002-S21F2 is not involved in recognizing the antigenic sites (Fig. 2), further indicating that footprints of such bnAbs may provide a template to guide rational vaccine design.

The structural and genetic analysis of 002-S21F2 bnAb shows that it is distinct from the previously reported SARS-CoV-2 mAbs. The cryo-EM structures of 002-S21F2 IgG with both WA.1 and Omicron provide a mechanistic rationale for its resilience against Omicron (BA.1, BA.2, BA.2.12.1, BA.4, and BA.5) possibly owing to unique molecular signatures that target a non-ACE2 binding conserved RBD epitope. Thus, 002-S21F2 bnAb has tremendous potential to treat patients with COVID-19. Together, the discovery and structural analysis of the bnAb 002-S21F2 and the broadly neutralizing epitope it targets provide valuable insights into immune mechanisms permitting potent neutralization of highly transmissible and immune evasive SARS-CoV-2 VOCs including the most recent Omicron sublineages.

## MATERIALS AND METHODS

### Human subjects

The International Centre for Genetic Engineering and Biotechnology (ICGEB) and Indian Council of Medical Research-National Institute of Malaria Research (ICMR-NIMR) institutional ethical boards approved the study. Informed consent was obtained from study subjects before inclusion in the study. COVID-19–recovered individuals have been described earlier (*15*). Of these, five subjects chosen based on the frequency of receptor binding protein–positive memory B cells and the available number of banked peripheral blood mononuclear cells (PBMCs) were included in this study for human mAb generation.

### SARS-CoV-2 RBD-specific ELISA binding assays

The recombinant SARS-CoV-2 RBD gene was cloned, expressed, and purified, and ELISAs were performed as previously described (*29*). Briefly, purified RBD was coated on MaxiSorp plates (Thermo Fisher Scientific, #439454) at a concentration of 1 μg/ml in phosphate-buffered saline (PBS) at 4°C overnight. The plates were washed extensively with PBS containing 0.05% Tween 20 (PBST). Three-fold serially diluted plasma or purified mAb was added to the plates and incubated at room temperature for 1 hour. After incubation, the plates were washed, and the SARS-CoV-2 RBD-specific IgG, IgM, and IgA signals were detected by incubating with horseradish peroxidase (HRP)–conjugated anti-human IgG (Jackson ImmunoResearch Laboratories, #109-036-098), IgM (Jackson ImmunoResearch Laboratories, #109-036-129), or IgA (Jackson ImmunoResearch Laboratories, #109-036-011). Plates were then washed thoroughly and developed with *o*-phenylenediamine substrate (Sigma-Aldrich, #P8787) in 0.05 M phosphate-citrate buffer (Sigma-Aldrich, #P4809) pH 5.0, containing 0.012% hydrogen peroxide (Thermo Fisher Scientific, #18755). Absorbance was measured at 490 nm.

### Authentic live SARS-CoV-2 neutralization assay

Neutralization titers to SARS-CoV-2 were determined as previously described (*15*, *29*). Briefly, 100 plaque-forming units of SARS-CoV-2 (2019-nCoV/USA_WA1/2020), Alpha, Beta, Gamma, Delta, and Omicron (BA.1, BA.2, BA.2.12.1, BA.4, and BA.5) was used on Vero-TMPRSS2 cells. The GISAID (global initiative on sharing all influenza data) IDs of Omicron live viral isolates used in neutralization assays are EPI_ISL_7171744 (BA.1), EPI_ISL_8818457 (BA.2), EPI_ISL_11685455 (BA.2.12.1), EPI_ISL_12416220 (BA.4), and EPI_ISL_13512579 (BA.5). Purified mAb was serially diluted threefold in duplicate starting at 10 μg/ml concentration in a 96-well round-bottom plate and incubated for 1 hour at 37°C. This antibody-virus mixture was transferred into the wells of a 96-well plate that had been seeded with Vero-TMPRSS2 cells the previous day at a concentration of 2.5 × 10^4^ cells per well. After 1 hour, the antibody-virus inoculum was removed, and 0.85% methylcellulose in 2% fetal bovine serum (FBS) containing Dulbecco’s minimal essential medium was overlaid onto the cell monolayer. Cells were incubated at 37°C for 16 to 40 hours. Cells were washed three times with 1× PBS (Corning cellgro) and fixed with 125 μl of 2% paraformaldehyde in PBS (Electron Microscopy Sciences) for 30 min. Following fixation, plates were washed twice with PBS, and 100 μl of permeabilization buffer was added to the fixed cells for 20 min. Cells were incubated with an anti–SARS-CoV spike primary antibody directly conjugated with Alexa Fluor 647 (CR3022-AF647) for up to 4 hours at room temperature.

Plates were then washed twice with 1× PBS and imaged on an ELISPOT reader (CTL Analyzer). Foci were counted using Viridot (counted first under the “green light” setting followed by background subtraction under the “red light” setting). IC_50_ titers were calculated by nonlinear regression analysis using the 4PL (four parameter logic) sigmoidal dose curve equation on Prism 9 (GraphPad Software). Neutralization titers were calculated as 100% × [1 − (average foci in duplicate wells incubated with the specimen) ÷ (average number of foci in the duplicate wells incubated at the highest dilution of the respective specimen)].

### SARS-CoV-2 RBD-specific memory B cell staining and single-cell sorting

Purified SARS-CoV-2 RBD protein was labeled with Alexa Fluor 488 using a microscale protein labeling kit (Life Technologies, #A30006) as per the manufacturer’s protocol. Ten million PBMCs of select COVID-19–recovered donors were stained with RBD–Alexa Fluor 488 for 1 hour at 4°C, followed by washing with PBS containing 2% FBS [fluorescence-activated cell sorting (FACS) buffer] and incubation with eFluor 780 Fixable Viability (Live/Dead) dye (Life Technologies, #65-0865-14) and anti-human CD3, CD19, CD20, CD27, CD38, and IgD antibodies (BD Biosciences) for 30 min. Cells were washed twice with FACS buffer and acquired on BD FACSAria Fusion (BD Biosciences). Live IgD-negative B cells that were positive for the SARS-CoV-2 RBD–Alexa Fluor 488 protein were single-cell–sorted into a 96-well plate containing 5 μl of lysis buffer. The lysis buffer consisted of 20 U of ribonuclease inhibitor (Promega) in 10 mM tris (pH 8.0) buffer. The plates with the sorted single cells were centrifuged gently at 2000 rpm at 4°C and stored immediately at −80°C for at least 1 hour before performing the complementary DNA (cDNA) synthesis. Data were analyzed using FlowJo software 10.

### Antibody gene amplification and cloning

The antibody genes were amplified and cloned as described earlier (*30*–*32*). Briefly, cDNA was synthesized, and antibody variable gene VDJ segments were amplified by reverse transcription polymerase chain reaction (PCR) using a template-switching rapid amplification of cDNA ends approach. Gene segments were cloned into AbVec6W vectors (*30*). Four colonies from each transformed plate were randomly picked, and the insert was checked by performing colony PCR using nested PCR primers. The sequence integrity of the plasmids was verified by Sanger Sequencing (Macrogen Sequencing, South Korea).

### Immunogenetic analyses of antibody genes

The immunogenetic analysis of both HC and LC germline assignment, framework region annotation, determination of SHM levels (nucleotides), and CDR loop lengths (amino acids) was performed with the aid of IMGT/HighV-QUEST (www.imgt.org/HighV-QUEST) (*33*).

### Expression of human monoclonal antibodies

For small-scale transfection, expi293F cells were maintained in 293 expression medium and transfected at a density of 2.5 million cells/ml in a volume of 4-ml culture per well of a six-well cell culture plate (Corning). The transfection mix consisted of a 1:1.5 HC/LC ratio using a 1:3 ratio with PEI-Max transfection reagent (1 mg/ml; Polysciences) in 200 μl of Opti-MEM. After 15-min incubation at room temperature, the transfection mix was added to the cells. Supernatants were harvested 4 to 5 days after transfection, and clarified supernatants were tested for their SARS-CoV-2 RBD binding potential by ELISA. The supernatant with positive RBD binding signals was next purified using protein A/G beads (Thermo Fisher Scientific), concentrated using a 30- or 100-kDa cutoff concentrator (Vivaspin, Sartorius), and stored at 4°C for further use.

### SARS-CoV-2 surrogate virus neutralization test

The potential of human ACE2 (hACE2) and SARS-CoV-2 RBD interaction inhibition by RBD-specific mAbs was measured with the cPass SARS-CoV-2 surrogate virus neutralization test kit (GenScript, Singapore) as described previously (*34*), as per the manufacturer’s protocol. Briefly, each mAb at a concentration of 20 μg/ml was mixed with equal volumes of recombinant HRP-conjugated RBD and incubated for 30 min at 37°C. Next, 100 μl of this mixture was transferred to 96-well plates coated with recombinant hACE2 receptor and further incubated for 15 min at 37°C. The plate was washed four times with 1× PBST buffer followed by the addition of tetramethylbenzidine substrate. The plate was incubated for 15 min at room temperature, and the reaction was stopped by adding the stop solution. Absorbance was measured at 450 nm, and the percentage of inhibition of each sample was calculated using the following formula: % inhibition = (1 − (OD_450_ sample/OD_450_ of negative control)) × 100. Controls were included in duplicate; samples were analyzed in the singular. Inhibition of >30% was regarded as a positive neutralization.

### Electrochemiluminescence antibody binding assay

Binding analysis of SARS-CoV-2 mAb to spike protein was performed using an electrochemiluminescence assay as previously described (*35*). V-PLEX COVID-19 Panel 24 (MSD) was used to measure the IgG1 mAb binding to SARS-CoV-2 spike antigens following the manufacturer’s recommendations. Briefly, antigen-coated plates were blocked with 150 μl per well of 5% bovine serum albumin in PBS for 30 min. Plates were washed three times with 150 μl per well of PBST between each incubation step. mAbs were serially diluted for concentrations ranging from 10 μg/ml to 0.1 pg/ml, and 50 μl per well was added to the plate and incubated for 2 hours at room temperature with shaking at 700 rpm. mAb antibody binding was then detected with 50 μl per well of MSD SULFO-TAG anti-human IgG antibody (diluted 1:200) incubated for 1 hour at room temperature with shaking at 700 rpm. A total of 150 μl per well of MSD Gold Read Buffer B was then added to each plate immediately before reading on an MSD QuickPlex plate reader.

### Octet BLI analysis

Octet BLI was performed using an Octet Red96 instrument (ForteBio Inc.) as described previously (*36*). Briefly, 5 μg/ml concentration of 002-S21F2 was captured on a protein A sensor, and its binding kinetics were tested with serial twofold-diluted RBD (600 to 37.5 nM) and spike HexaPro protein (100 to 6.25 nM). The baseline was obtained by measurements taken for 60 s in BLI buffer (1× PBST), and then the sensors were subjected to association phase immersion for 300 s in wells containing serial dilutions of RBD or trimeric spike HexaPro protein. Then, the sensors were immersed in BLI buffer for as long as 600 s to measure the dissociation phase. The mean *K*_on_, *K*_off_, and apparent affinity constant (*K*_D_) values of the mAbs binding affinities for RBD and spike HexaPro were calculated from all the binding curves based on their global fit to a 1:1 Langmuir binding model using Octet software version 12.0.

### Spike protein expression and purification

SARS-CoV-2 spike-6P trimer protein carrying WA.1 and Omicron strain mutations was produced by transfecting FreeStyle 293-F cells using WA.1 spike-6P and Omicron spike-6P DNA plasmids, respectively. There are two mismatched amino acid positions in our Omicron plasmid: (i) positions 213 to 216 in the N-terminal domain are EPER instead of sequence REPE and (ii) position 493 is a lysine residue instead of an arginine. Transfections were performed as per the manufacturer’s protocol (Thermo Fisher Scientific). Briefly, FreeStyle 293-F cells were seeded at a density of 2 × 10^6^ cells/ml in Expi293 Expression Medium and incubated at 37°C and 127 rpm with 8% CO_2_ overnight. The next day, 2.5 × 10^6^ cells/ml was transfected using ExpiFectamine 293 transfection reagent (Thermo Fisher Scientific, catalog no. A14524). The cells continued to grow for 4 to 5 days at 37°C, 127 rpm, 8% CO_2_ incubator. The cells were removed by centrifugation at 4000*g* for 20 min at room temperature, and the spike protein–containing supernatant was collected. The supernatant was filtered and loaded onto prewashed His-Pur Ni-NTA (nickel–nitrilotriacetic acid) resin for affinity purification. The Ni-NTA resin was incubated with a spike trimer–containing supernatant for 2 hours on a shaker at room temperature. Resin was washed with wash buffer containing 25 mM imidazole, 6.7 mM NaH_2_PO_4_·H_2_O, and 300 mM NaCl in PBS followed by spike protein elution in elution buffer containing 235 mM imidazole, 6.7 mM NaH_2_PO_4_·H_2_O, and 300 mM NaCl in PBS. Eluted protein was dialyzed against PBS and was concentrated. The concentrated protein ran onto a Superose-6 Increase 10/300 column, and protein was eluted as trimeric spike was collected. The quality of the protein was evaluated by SDS–polyacrylamide gel electrophoresis and by negative stain EM.

### Negative stain electron microscopy

Spike protein was diluted to 0.05 mg/ml in PBS before grid preparation. A 3-μl drop of diluted protein was applied to previously glow-discharged, carbon-coated grids for ~60 s, blotted and washed twice with water, stained with 0.75% uranyl formate, blotted, and air-dried. Between 30 and 50 images were collected on a Talos L120C microscope (Thermo Fisher Scientific) at ×73,000 magnification and a pixel size of 1.97 Å. RELION 3.1 (*37*) or cryoSPARC v3.3.2 (*38*) was used for particle picking, two-dimensional (2D) classification.

### Sample preparation for cryo-EM

SARS-CoV-2 spike-6P trimer was incubated with the mAb (full-length IgG) at a concentration of 0.7 mg/ml. The complex was prepared at a 0.4 submolar ratio of mAb to prevent interspike cross-linking, mediated by bivalent binding of Fab in IgG. The complex was incubated at room temperature for ~5 min before vitrification. Three microliters of the complex was applied onto a freshly glow-discharged (PELCO easiGLOW) 400-mesh, 1.2/1.3 C-Flat grid (Electron Microscopy Sciences). After 20 s of incubation, grids were blotted for 3 s at 0 blot force and vitrified using a Vitrobot IV (Thermo Fisher Scientific) under 22°C with 100% humidity.

### Cryo-EM data acquisition

Single-particle cryo-EM data for mAb 002-S21F2 in complex with WA.1 and Omicron spike-6p protein were collected on a 200-kV Talos Arctica transmission electron microscope (Thermo Fisher Scientific) equipped with a Gatan K3 direct electron detector behind a 20-eV slit width energy filter. Multiframe movies were collected at a pixel size of 1.1 Å per pixel with a total dose of 51 e/Å^2^ at defocus range of −1.0 to −2.4 μm.

### Cryo-EM data analysis and model building

Cryo-EM movies were motion-corrected either in MotionCorr2 in RELION 3.0 (*37*, *39*) or using Patch motion correction implemented in cryoSPARC v3.3.1 (*38*). When Motion correction was performed outside of cryoSPARC, motion-corrected micrographs were imported in cryoSPARC v3.3.1 and corrected for contrast transfer function using cryoSPARC’s implementation of Patch contrast transfer function (CTF) estimation. Micrographs with poor CTF fits were discarded using CTF fit resolution cutoff to ~6.0 Å. Particles were picked using a Blob picker, extracted, and subjected to an iterative round of 2D classification. Particles belonging to the best 2D classes with secondary structure features were selected for heterogeneous 3D refinement to separate IgG-bound spike particles from non–IgG-bound spike particles. Particles belonging to the best IgG-bound 3D class were refined in nonuniform 3D refinement with per particle CTF and higher-order aberration correction turned on. To further improve the resolution of the RBD-IgG binding interface, a soft mask was created covering one RBD and interacting Fab region of IgG and refined locally in cryoSPARC using local refinement on signal subtracted particles. All maps were density-modified in Phenix (*40*) using resolve cryo-EM. The combined Focused Map tool in Phenix was used to integrate high-resolution locally refined maps into an overall map. Additional data processing details are summarized in figs. S4 and S5.

The initial spike models for WA.1 (PDB:7lrt) or Omicron (PDB:7tf8) as well as individual HC and LC of the Fab region of an IgG [generated with Alphafold (*41*)] were docked into combined focused cryo-EM density maps using UCSF ChimeraX (*42*). The full spike mAb model was refined using rigid body refinement in Phenix, followed by refinement in Isolde (*43*). The final model was refined further in Phenix using real-space refinement. Glycans with visible density were modeled in Coot and validated by Privateer (*44*, *45*). Model validation was performed using MolProbity (*46*). PDBePISA (*47*) was used to identify mAb-RBD interface residue to calculate buried surface area and to identify polar interaction. Figures were prepared in ChimeraX (*42*) and PyMOL (*48*).
