# Supplementary material for: Structural insights for neutralization of Omicron variants BA.1, BA.2, BA.4, and BA.5 by a broadly neutralizing SARS-CoV-2 antibody
Source: Sci Adv. 2022 Oct 5;8(40):eadd2032. doi: 10.1126/sciadv.add2032 (PMC9534492; doi:10.1126/sciadv.add2032)
Supplement: Supplementary file 1 — Figs. S1 to S9 Tables S1 to S3 [file sciadv.add2032_sm.pdf]

Science Advances
AAAS

Supplementary Materials for

Structural insights for neutralization of Omicron variants BA.1, BA.2, BA.4, and BA.5 by a broadly neutralizing SARS-CoV-2 antibody

Sanjeev Kumar *et al.*

Corresponding author: chandeleanmol@gmail.com, Anmol Chandele, anmol@icgeb.res.in;
Kaja Murali-Krishna, murali.kaja@emory.edu; Amit Sharma, directornimr@gmail.com;
Eric Ortlund, eortlun@emory.edu

Sci. Adv. **8**, eadd2032 (2022)
DOI: 10.1126/sciadv.add2032

This PDF file includes:

Figs. S1 to S9
Tables S1 to S3

Supplementary Figures

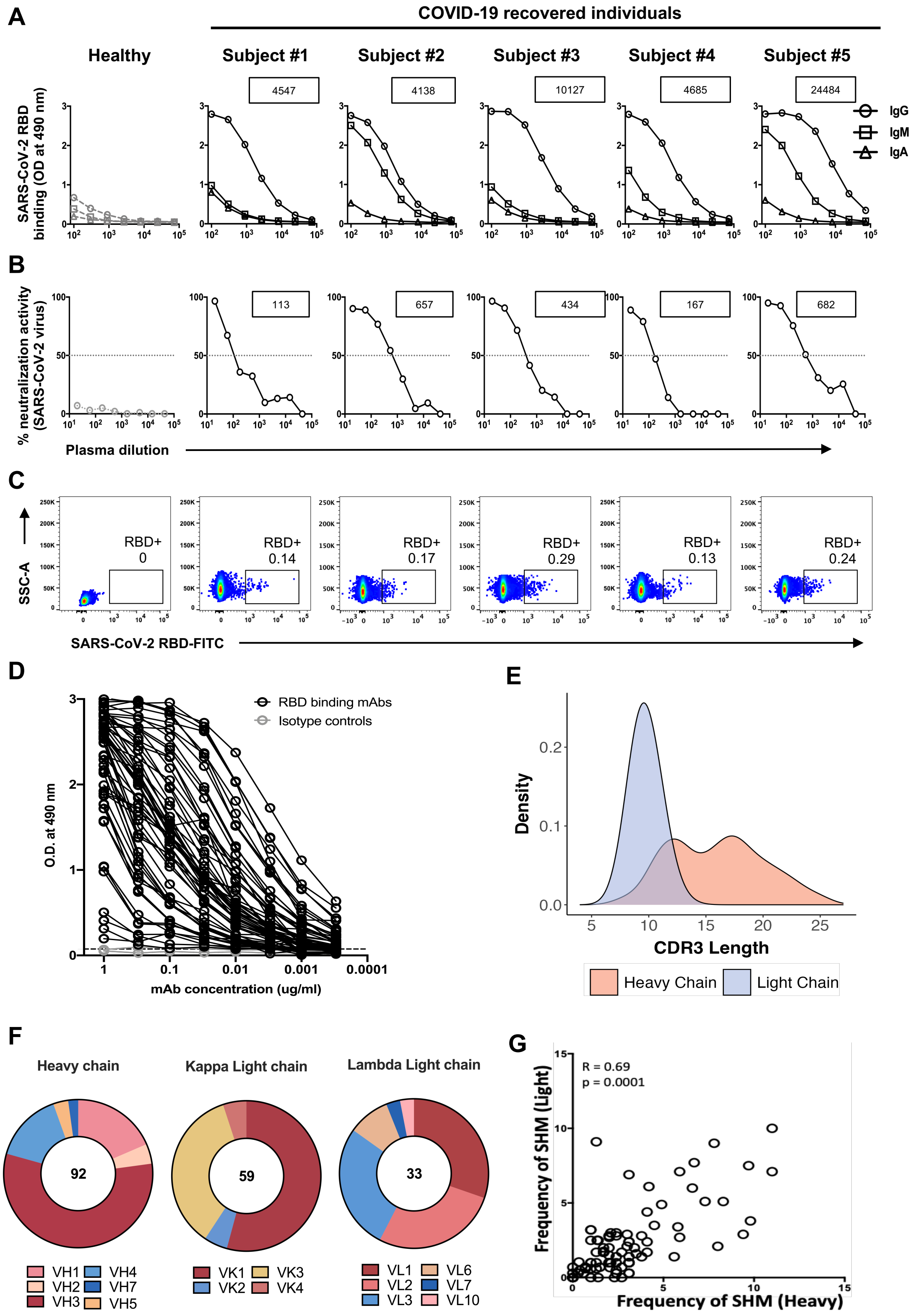

Fig. S1: Isolation and characterization of 92 SARS-CoV-2 RBD specific mAbs. (A) ELISA curves showing plasma IgG, IgM and IgA reactivity against SARS-CoV-2 RBD protein. The five subjects are COVID-19 recovered individuals recruited into a clinical study during the first wave of the pandemic in India. ELISA results with the plasma of a pre-pandemic healthy individual are shown for comparison. RBD-specific Max_{50} IgG binding titers for are shown in boxes. **(B)** Neutralization assay curves showing activity against ancestral SARS-CoV-2 Wuhan-I (WA.1) strain. The plasma dilution resulting in a 50% reduction in neutralization ($FRNT\text{-}mNG_{50}$) is indicated in the boxes. **(C)** Flow cytometry plots show SARS-CoV-2 RBD-specific memory B cells in the peripheral blood lymphocytes of select SARS-CoV-2 recovered individuals. The plots shown were gated on CD19 positive and CD3 negative populations. Staining was performed using FITC conjugated RBD protein. **(D)** ELISA curves showing SARS-CoV-2 RBD binding by each of the purified mAbs (n=92). **(E)** The CDR3 length of each sequence was calculated and their distribution was plotted as a histogram. **(F)** Donut plots showing the heavy chain, lambda light chain and kappa light chain gene distribution of the SARS-CoV-2 RBD-specific mAbs (n=92). **(G)** Correlation plot of heavy chain and light chain SHM (%). Pearson correlation coefficient (R) and p-value have been shown.

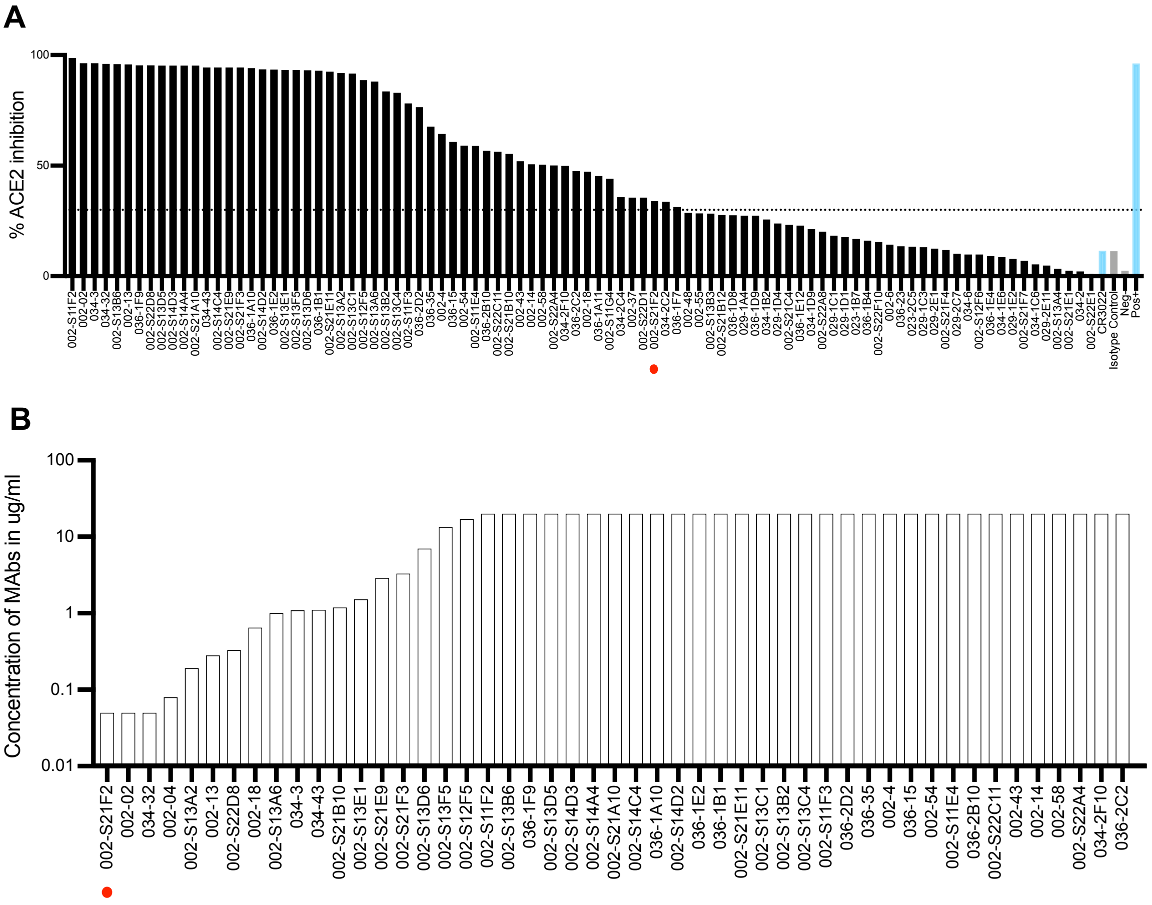

Fig. S2: ACE2 inhibition and neutralization potential analysis of 92 SARS-CoV-2 RBD specific mAbs. (A) ACE2 inhibition analysis of SARS-CoV-2 RBD specific human monoclonal antibodies (n=92) was performed using the cPass™ SARS-CoV-2 surrogate neutralization antibody detection kit (Genscript, USA). All mAbs were tested at 20 µg/ml. Percent (%) inhibition values are plotted as a bar diagram (Black colour). Here, CR3022, a SARS-CoV and SARS-CoV-2 specific mAb was used as a positive control, A5GK (an inhouse CHIKV specific mAb) and D2-DV (an inhouse DENV mAb) were used as negative isotype control mAbs. The dotted line of the 30% cut-off value shows the set threshold over which ACE2 inhibition is positive as per manufacturers' instructions. **(B)** Neutralization titers (FRNT-mNG$_{50}$) against SARS-CoV-2 live virus (stain (WA.1/2020) of the few RBD binding mAbs. Here, mAb 002-S21F2 is marked with a red dot.

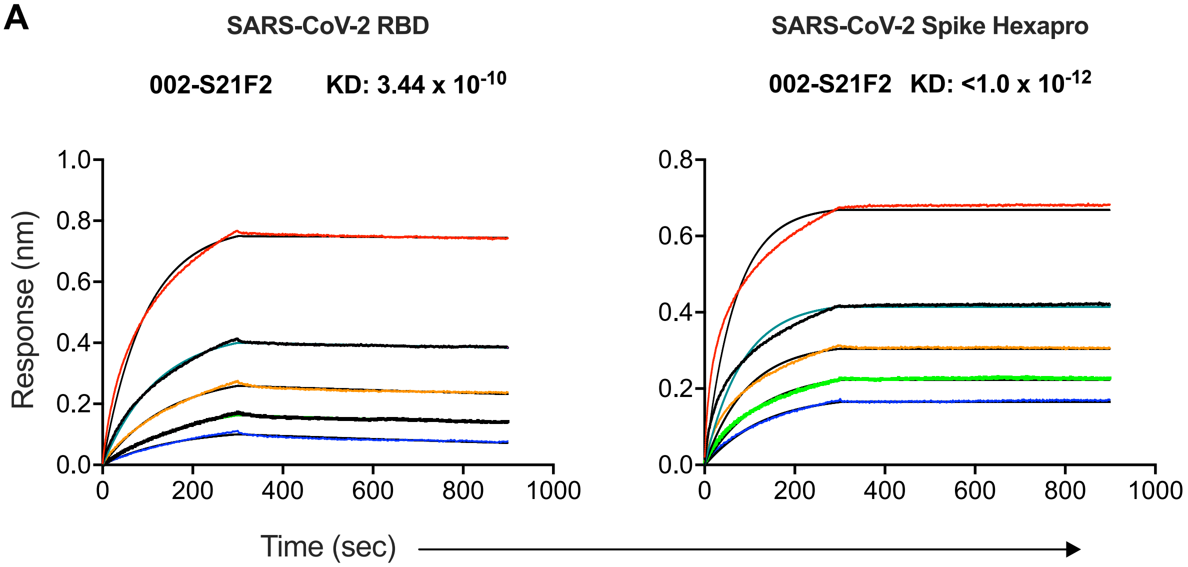

B

	SARS-CoV-2 WT RBD			SARS-CoV-2 WT Spike hexapro		
**mAb**	**KD (M)**	**K_{on} (1/Ms)**	**K_{off} (1/s)**	**KD (M)**	**K_{on} (1/Ms)**	**K_{off} (1/s)**
**002-S21F2**	3.44E-10	5.44E+05	2.18E-04	<1.0E-12	8.05E+05	<1.0E-07

Fig. S3: Antibody 002-S21F2 showed high affinity to SARS-CoV-2 RBD and spike proteins in biolayer interferometry assays. (A) Octet BLI sensorgrams showing the SARS-CoV-2 RBD and Spike-6p binding affinities of the mAb 002-S21F2. In these assays, 002-S21F2 mAb (5 ug/ml concentration) was captured on protein A sensors and its binding kinetics were tested with serial 2-fold diluted RBD (600 nM to 37.5 nM) and Spike hexapro protein (100 nM to 6.25 nM). Association was measured for 300 seconds followed by dissociation measurement for 600 seconds. **(B)** Describing the KD (M), K_{on} (1/Ms) and K_{off} (1/s) values of the four potent mAbs with RBD and Spike hexapro proteins.

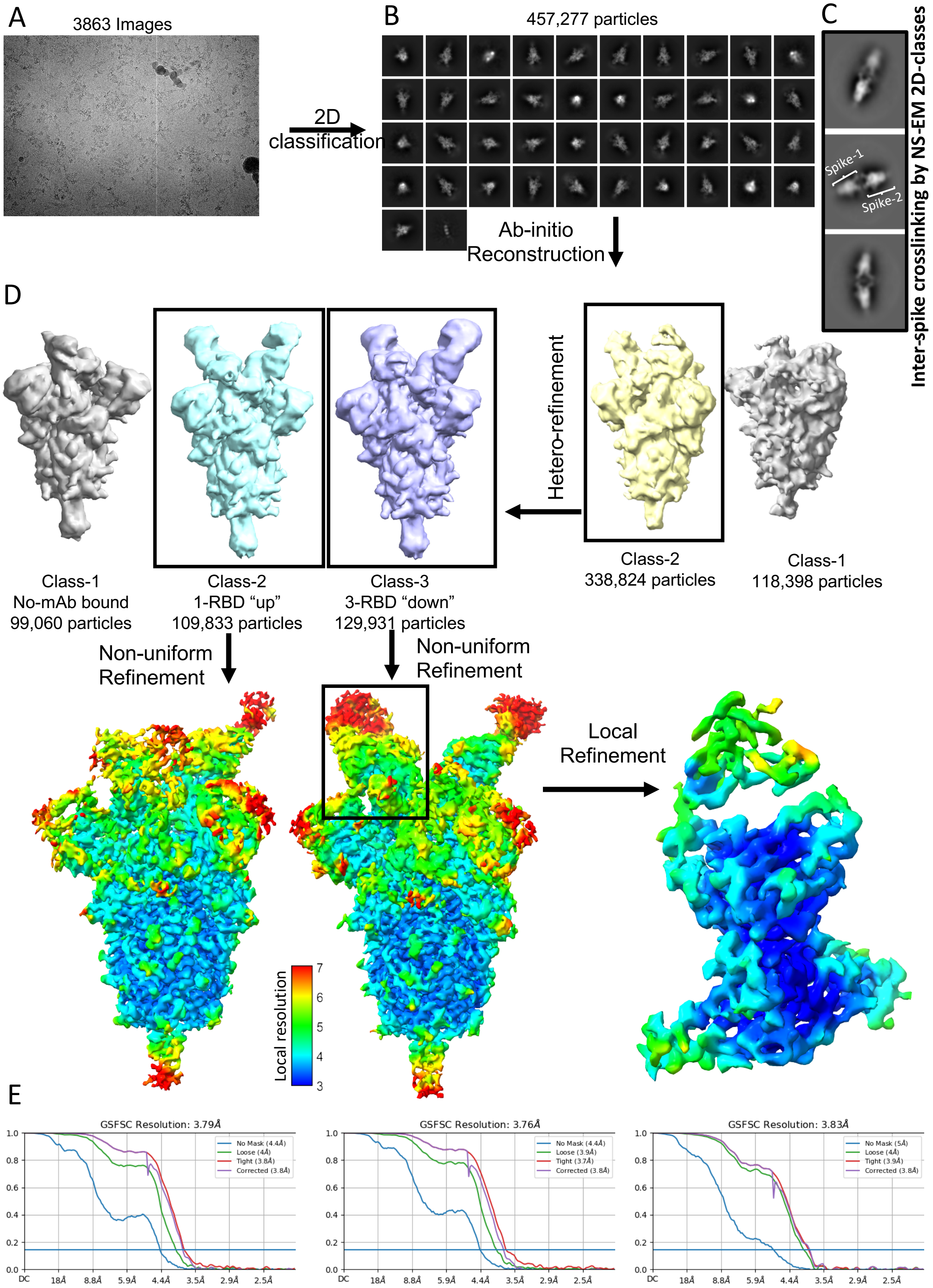

A
3863 Images
2D
classification
B
457,277 particles
Ab-initio
Reconstruction
C
Spike-1
Spike-2
Inter-spike crosslinking by NS-EM 2D-classes
D
Hetero-refinement
Class-1
No-mAb bound
99,060 particles
Class-2
1-RBD “up”
109,833 particles
Class-3
3-RBD “down”
129,931 particles
Class-2
338,824 particles
Class-1
118,398 particles
Non-uniform
Refinement
Non-uniform
Refinement
Local
Refinement
Local resolution
E
GSFSC Resolution: 3.79Å
GSFSC Resolution: 3.76Å
GSFSC Resolution: 3.83Å

Fig. S4. CryoEM data analysis and validation for WA.1 Spike-6P 002-S21F2 complex. **(A)** Representative electron micrograph. **(B)** Representative 2D-class averages. **(C)** The 2D-Classes derived from Negative Stain EM (NS-EM) data of WA.1 Spike-6P and 002-S21F2 complex at saturating IgG concentration show head-to-head spike dimer. **(D)** Classification scheme and refinement that yielded final cryoEM map reconstruction. Boxed classes were selected for further processing and refinement. The boxed region contains one RBD complexed with one Fab in a refined map masked for local refinement**.** **(E)** Gold standard Fourier shell correlation curve of final overall (two left panels) and locally refined (right panel) maps and resolution estimation based on 0.143 Fourier shell correlation criteria as indicated by a blue line.

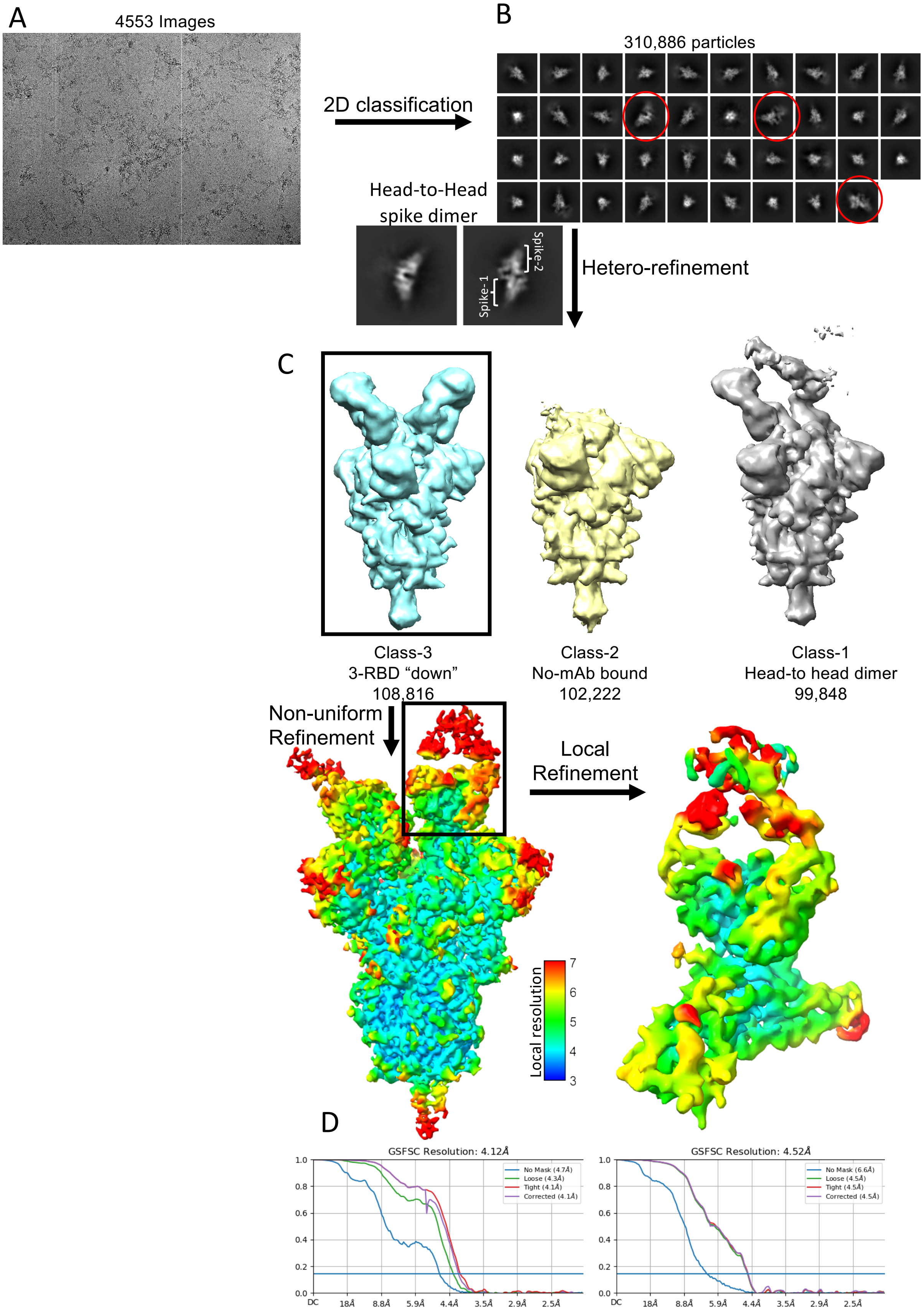
A
4553 Images
2D classification
B
310,886 particles
Head-to-Head
spike dimer
Spike-2
Spike-1
Hetero-refinement
C
Class-3
3-RBD "down"
108,816
Class-2
No-mAb bound
102,222
Class-1
Head-to head dimer
99,848
Non-uniform
Refinement
Local
Refinement
Local resolution
7
6
5
4
3
D
GSFSC Resolution: 4.12Å
No Mask (4.7Å)
Loose (4.3Å)
Tight (4.1Å)
Corrected (4.1Å)
GSFSC Resolution: 4.52Å
No Mask (6.6Å)
Loose (4.5Å)
Tight (4.5Å)
Corrected (4.5Å)
DC
18Å
8.8Å
5.9Å
4.4Å
3.5Å
2.9Å
2.5Å

Fig. S5. Cryo-EM data analysis and validation for Omicron Spike-6P and 002-S21F2 complex. (A) Representative electron micrograph. **(B)** Representative 2D-class averages. 2D classes showing head-to-head spike dimer are circled in red and shown below. **(C)** Classification scheme and refinement that yielded final cryo-EM map reconstruction. Boxed classes were selected for further processing and refinement. Boxed region containing one RBD complexed with one Fab in refined map masked for local refinement. **(D)** Gold standard Fourier shell correlation (FSC) curve of final overall (left) and locally refined (right) maps and resolution estimation based on 0.143 Fourier shell correlation criteria as indicated by a blue line.

A

	Heavy Chain						Light Chain				
mAbs	V-gene	D-gene	J-gene	CDRH3	CDRH3 Length	SHM (%)	V-gene	J-gene	CDRH3	CDRL3 Length	SHM (%)
002-S21F2	IGHV5-51*01	IGHD5-24*01	IGHJ4*02	ARGEMTAVFGDY	12	2.7	IGKV1-33*01	IGKJ2*01	QQYKILLTWT	10	1.7

B

```
             FR1-IMGT                CDR1-IMGT      FR2-IMGT           CD
             (1-26)                  (27-38)        (39-55)            (
             1         10        20          30           40        50
             .........|.........|......  ...|........ .|.........|..... ..
002-S21F2    EVQLVQSGA.EVKKPGESLKISCKAS  GYNF....AYYW IGWVRQMPGKGLEWMGI IY
IGHV5-51 gl  EVQLVQSGA.EVKKPGESLKISCKGS  GYSF....TSYW IGWVRQMPGKGLEWMGI IY
                                     A     N     AY

             R2-IMGT          FR3-IMGT
             56-65)           (66-104)
              60        70        80        90        100
             ..|.....  ....|.........|.........|.........|....
             PG..DSDT  RYSPSFQ.GQVTISADKSISTAYLQWSSLKASDTAMYYC AR
             PG..DSDT  RYSPSFQ.GQVTISADKSISTAYLQWSSLKASDTAMYYC AR
```

```
             FR1-IMGT                CDR1-IMGT      FR2-IMGT           CD
             (1-26)                  (27-38)        (39-55)            (
             1         10        20          30           40        50
             .........|.........|......  ...|........ .|.........|..... ..
002-S21F2    DIQMTQSPSSLSASVGDRVTITCQAS  QDI......SNY LNWYQQKPGKAPKLLIY DA
IGKV1-33 gl  DIQMTQSPSSLSASVGDRVTITCQAS  QDI......SNY LNWYQQKPGKAPKLLIY DA

             R2-IMGT          FR3-IMGT
             56-65)           (66-104)
              60        70        80        90        100
             ..|.....  ....|.........|.........|.........|....
             .......S  NLETGVP.SRFSGSG..SGTDFTLTISSLQPEDIATYYC QQYKIL
             .......S  NLETGVP.SRFSGSG..SGTDFTFTISSLQPEDIATYYC QQYDNLP
                                          L                     KI
```

C

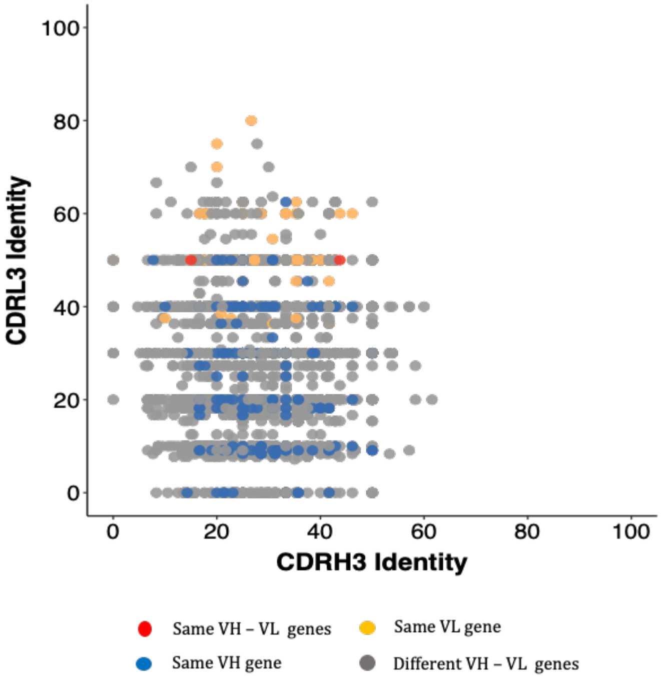

Fig. S6. SARS-CoV-2 RBD specific bnAb 002-S21F2 exhibits a rare immunogenetic make-up. **(A)** Table showing the antibody heavy chain (HC) and light chain (LC) genetic information of the 002-S21F2 bnAb. **(B)** IMGT V-quest alignment analysis result of 002-S21F2 HC and LC gene with their respective germline sequence. Here, somatic mutations are marked as bold amino acids in black color. **(C)** CDRH3 amino acid sequence identity to 002-S21F2 is plotted against CDRL3 amino acid identity to 002-S21F2 for paired HC and LC sequences of SARS-CoV-2 mAbs banked in the CoV-AbDab database. SARS-CoV-2 mAbs with the same HC and LC germline gene as 002-S21F2 (IGHV5-51 and IGKV1-33) are shown in red color. MAbs using the same HC gene but different LC gene as 002-S21F2 are shown in blue color. MAbs using different HC genes but the same LC gene as 002-S21F2 are shown in orange color. MAbs using a different HC and LC combination as 002-S21F2 are shown in grey color.

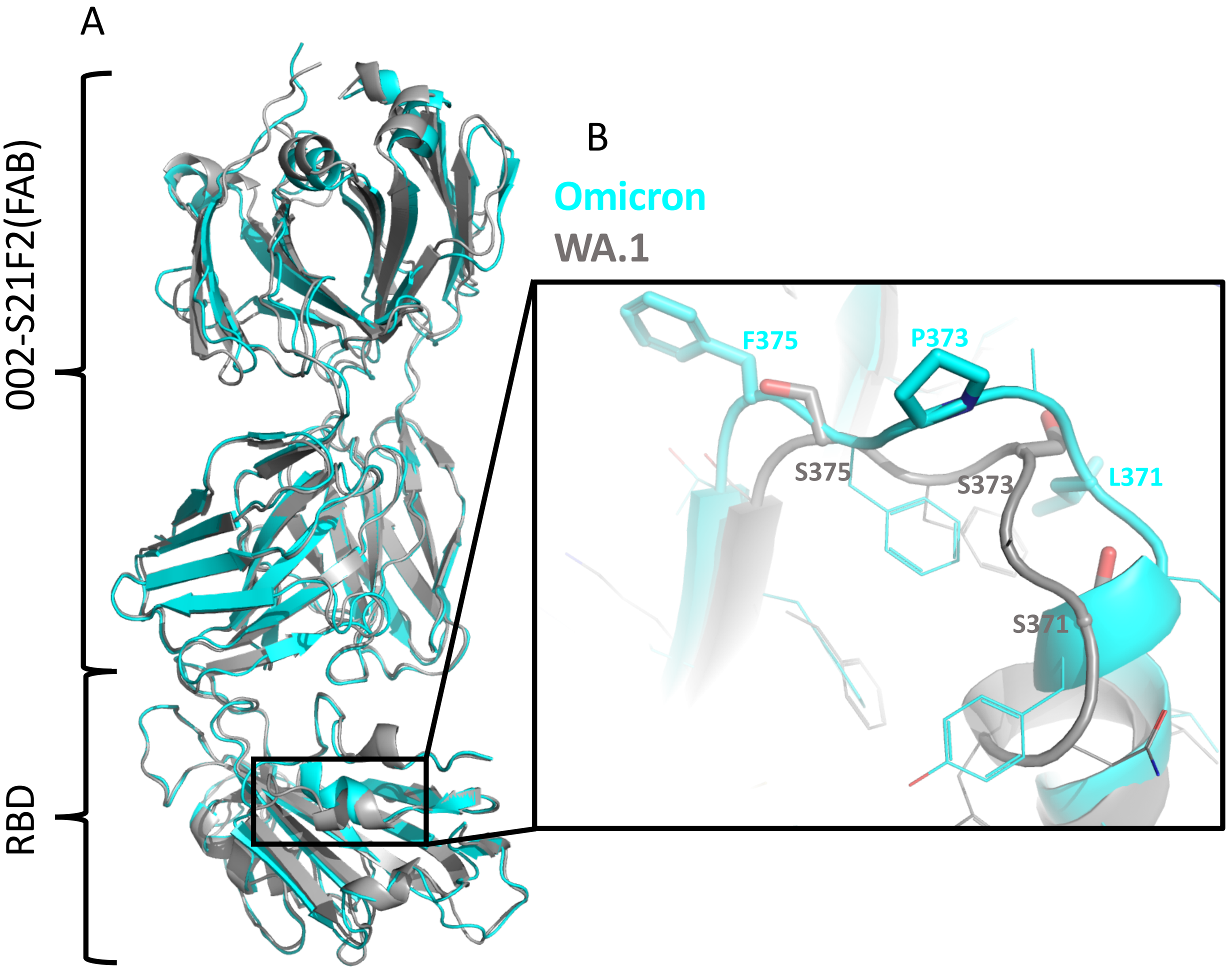

Fig. S7. Structural comparison of WA.1 (grey) and Omicron (cyan) spike-002-S21F2 mAb complex. (A) Overall structural alignment of RBD and Fab region of 002-S21F2 in WA.1 and Omicron complex structures. **(B)** Zoomed in view showing the local conformation change in the specified region of RBD in WA.1 vs Omicron.

		RBD mutations																						
SARS-CoV-2 variants		G339D	R346K	S371L/F	S373P	S375F	T376A	D405N	R408S	K417N/T	N440K	G446S	L452R/Q/M	N460K	S477N	T478K	E484K/Q/A	F486V	F490S	Q493R	G496S	Q498R	N501Y	Y505H
Alpha	B.1.1.7																							
Beta	B.1.351									N							K							
Gamma	P.1									T							K							
Delta	B.1.617.2												R											
Epsilon	B.1.427/9												R											
Zeta	P.2																K							
Eta	B.1.525																K							
Theta	P.3																K							
Iota	B.1.526																K							
Kappa	B.1.617.1												R				Q							
Lambda	C.37												Q											
Mu	B.1.621																K							
Omicron	B.1.1.529			L													A							
	BA.2			F													A							
	BA.2.12.1			F									Q				A							
	BA.2.75	H		F													A							
	BA.3			F													A							
	BA.4/BA.5			F									R				A							

Fig. S8. Represents the SARS-CoV-2 RBD mutations reported in key variants. The 002-S21F2 epitope residues are highlighted in orange on RBD mutations. Green color highlighted boxes show the presence of RBD mutations in each SARS-CoV-2 variant.

```
Kappa           NITNLCPFGEVFNATRFASVYAWNRKRISNCVADYSVLYNSASFSTFKCYGVSPTKLNDL      390
Delta           NITNLCPFGEVFNATRFASVYAWNRKRISNCVADYSVLYNSASFSTFKCYGVSPTKLNDL      390
Epsilon         NITNLCPFGEVFNATRFASVYAWNRKRISNCVADYSVLYNSASFSTFKCYGVSPTKLNDL      390
Lambda          NITNLCPFGEVFNATRFASVYAWNRKRISNCVADYSVLYNSASFSTFKCYGVSPTKLNDL      390
Zeta            NITNLCPFGEVFNATRFASVYAWNRKRISNCVADYSVLYNSASFSTFKCYGVSPTKLNDL      390
Eta             NITNLCPFGEVFNATRFASVYAWNRKRISNCVADYSVLYNSASFSTFKCYGVSPTKLNDL      390
Iota            NITNLCPFGEVFNATRFASVYAWNRKRISNCVADYSVLYNSASFSTFKCYGVSPTKLNDL      390
WA.1            NITNLCPFGEVFNATRFASVYAWNRKRISNCVADYSVLYNSASFSTFKCYGVSPTKLNDL      390
Alpha           NITNLCPFGEVFNATRFASVYAWNRKRISNCVADYSVLYNSASFSTFKCYGVSPTKLNDL      390
Theta           NITNLCPFGEVFNATRFASVYAWNRKRISNCVADYSVLYNSASFSTFKCYGVSPTKLNDL      390
Gamma           NITNLCPFGEVFNATRFASVYAWNRKRISNCVADYSVLYNSASFSTFKCYGVSPTKLNDL      390
Mu              NITNLCPFGEVFNATKFASVYAWNRKRISNCVADYSVLYNSASFSTFKCYGVSPTKLNDL      390
Beta            NITNLCPFGEVFNATRFASVYAWNRKRISNCVADYSVLYNSASFSTFKCYGVSPTKLNDL      390
Omi-BA.1        NITNLCPFDEVFNATRFASVYAWNRKRISNCVADYSVLYNLAPFFTFKCYGVSPTKLNDL      390
Omi-BA.3        NITNLCPFDEVFNATRFASVYAWNRKRISNCVADYSVLYNLAPFFTFKCYGVSPTKLNDL      390
Omi-BA.2.75     NITNLCPFHEVFNATRFASVYAWNRKRISNCVADYSVLYNFAPFFAFKCYGVSPTKLNDL      390
Omi-BA.2        NITNLCPFDEVFNATRFASVYAWNRKRISNCVADYSVLYNFAPFFAFKCYGVSPTKLNDL      390
Omi-BA.2.12.1   NITNLCPFDEVFNATRFASVYAWNRKRISNCVADYSVLYNFAPFFAFKCYGVSPTKLNDL      390
Omi-BA.4        NITNLCPFDEVFNATRFASVYAWNRKRISNCVADYSVLYNFAPFFAFKCYGVSPTKLNDL      390
Omi-BA.5        NITNLCPFDEVFNATRFASVYAWNRKRISNCVADYSVLYNFAPFFAFKCYGVSPTKLNDL      390
                ******** ******:************************ * * :**************

Kappa           CFTNVYADSFVIRGDEVRQIAPGQTGKIADYNYKLPDDFTGCVIAWNSNNLDSKVGGNYN      450
Delta           CFTNVYADSFVIRGDEVRQIAPGQTGKIADYNYKLPDDFTGCVIAWNSNNLDSKVGGNYN      450
Epsilon         CFTNVYADSFVIRGDEVRQIAPGQTGKIADYNYKLPDDFTGCVIAWNSNNLDSKVGGNYN      450
Lambda          CFTNVYADSFVIRGDEVRQIAPGQTGKIADYNYKLPDDFTGCVIAWNSNNLDSKVGGNYN      450
Zeta            CFTNVYADSFVIRGDEVRQIAPGQTGKIADYNYKLPDDFTGCVIAWNSNNLDSKVGGNYN      450
Eta             CFTNVYADSFVIRGDEVRQIAPGQTGKIADYNYKLPDDFTGCVIAWNSNNLDSKVGGNYN      450
Iota            CFTNVYADSFVIRGDEVRQIAPGQTGKIADYNYKLPDDFTGCVIAWNSNNLDSKVGGNYN      450
WA.1            CFTNVYADSFVIRGDEVRQIAPGQTGKIADYNYKLPDDFTGCVIAWNSNNLDSKVGGNYN      450
Alpha           CFTNVYADSFVIRGDEVRQIAPGQTGKIADYNYKLPDDFTGCVIAWNSNNLDSKVGGNYN      450
Theta           CFTNVYADSFVIRGDEVRQIAPGQTGKIADYNYKLPDDFTGCVIAWNSNNLDSKVGGNYN      450
Gamma           CFTNVYADSFVIRGDEVRQIAPGQTGTIADYNYKLPDDFTGCVIAWNSNNLDSKVGGNYN      450
Mu              CFTNVYADSFVIRGDEVRQIAPGQTGNIADYNYKLPDDFTGCVIAWNSNNLDSKVGGNYN      450
Beta            CFTNVYADSFVIRGDEVRQIAPGQTGNIADYNYKLPDDFTGCVIAWNSNNLDSKVGGNYN      450
Omi-BA.1        CFTNVYADSFVIRGDEVRQIAPGQTGNIADYNYKLPDDFTGCVIAWNSNKLDSKVSGNYN      450
Omi-BA.3        CFTNVYADSFVIRGNEVRQIAPGQTGNIADYNYKLPDDFTGCVIAWNSNKLDSKVSGNYN      450
Omi-BA.2.75     CFTNVYADSFVIRGNEVSQIAPGQTGNIADYNYKLPDDFTGCVIAWNSNKLDSKVSGNYN      450
Omi-BA.2        CFTNVYADSFVIRGNEVSQIAPGQTGNIADYNYKLPDDFTGCVIAWNSNKLDSKVGGNYN      450
Omi-BA.2.12.1   CFTNVYADSFVIRGNEVSQIAPGQTGNIADYNYKLPDDFTGCVIAWNSNKLDSKVGGNYN      450
Omi-BA.4        CFTNVYADSFVIRGNEVSQIAPGQTGNIADYNYKLPDDFTGCVIAWNSNKLDSKVGGNYN      450
Omi-BA.5        CFTNVYADSFVIRGNEVSQIAPGQTGNIADYNYKLPDDFTGCVIAWNSNKLDSKVGGNYN      450
                **************:** ********.**********************:*****.****

Kappa           YRYRLFRKSNLKPFERDISTEIYQAGSTPCNGVQGFNCYFPLQSYGFQPTNGVGYQPYRV      510
Delta           YRYRLFRKSNLKPFERDISTEIYQAGSKPCNGVEGFNCYFPLQSYGFQPTNGVGYQPYRV      510
Epsilon         YRYRLFRKSNLKPFERDISTEIYQAGSTPCNGVEGFNCYFPLQSYGFQPTNGVGYQPYRV      510
Lambda          YQYRLFRKSNLKPFERDISTEIYQAGSTPCNGVEGFNCYSPLQSYGFQPTNGVGYQPYRV      510
Zeta            YLYRLFRKSNLKPFERDISTEIYQAGSTPCNGVKGFNCYFPLQSYGFQPTNGVGYQPYRV      510
Eta             YLYRLFRKSNLKPFERDISTEIYQAGSTPCNGVKGFNCYFPLQSYGFQPTNGVGYQPYRV      510
Iota            YLYRLFRKSNLKPFERDISTEIYQAGSTPCNGVKGFNCYFPLQSYGFQPTNGVGYQPYRV      510
WA.1            YLYRLFRKSNLKPFERDISTEIYQAGSTPCNGVEGFNCYFPLQSYGFQPTNGVGYQPYRV      510
Alpha           YLYRLFRKSNLKPFERDISTEIYQAGSTPCNGVEGFNCYFPLQSYGFQPTYGVGYQPYRV      510
Theta           YLYRLFRKSNLKPFERDISTEIYQAGSTPCNGVKGFNCYFPLQSYGFQPTYGVGYQPYRV      510
Gamma           YLYRLFRKSNLKPFERDISTEIYQAGSTPCNGVKGFNCYFPLQSYGFQPTYGVGYQPYRV      510
Mu              YLYRLFRKSNLKPFERDISTEIYQAGSTPCNGVKGFNCYFPLQSYGFQPTYGVGYQPYRV      510
Beta            YLYRLFRKSNLKPFERDISTEIYQAGSTPCNGVKGFNCYFPLQSYGFQPTYGVGYQPYRV      510
Omi-BA.1        YLYRLFRKSNLKPFERDISTEIYQAGNKPCNGVAGFNCYFPLRSYSFRPTYGVGHQPYRV      510
Omi-BA.3        YLYRLFRKSNLKPFERDISTEIYQAGNKPCNGVAGFNCYFPLRSYGFRPTYGVGHQPYRV      510
Omi-BA.2.75     YLYRLFRKSKLKPFERDISTEIYQAGNKPCNGVAGFNCYFPLQSYGFRPTYGVGHQPYRV      510
Omi-BA.2        YLYRLFRKSNLKPFERDISTEIYQAGNKPCNGVAGFNCYFPLRSYGFRPTYGVGHQPYRV      510
Omi-BA.2.12.1   YQYRLFRKSNLKPFERDISTEIYQAGNKPCNGVAGFNCYFPLRSYGFRPTYGVGHQPYRV      510
Omi-BA.4        YRYRLFRKSNLKPFERDISTEIYQAGNKPCNGVAGVNCYFPLQSYGFRPTYGVGHQPYRV      510
Omi-BA.5        YRYRLFRKSNLKPFERDISTEIYQAGNKPCNGVAGVNCYFPLQSYGFRPTYGVGHQPYRV      510
                * *******:****************..***** *.*** **:**.*:** ***:*****

Kappa           VVLSFE        516
Delta           VVLSFE        516
Epsilon         VVLSFE        516
Lambda          VVLSFE        516
Zeta            VVLSFE        516
Eta             VVLSFE        516
Iota            VVLSFE        516
WA.1            VVLSFE        516
Alpha           VVLSFE        516
Theta           VVLSFE        516
Gamma           VVLSFE        516
Mu              VVLSFE        516
Beta            VVLSFE        516
Omi-BA.1        VVLSFE        516
Omi-BA.3        VVLSFE        516
Omi-BA.2.75     VVLSFE        516
Omi-BA.2        VVLSFE        516
Omi-BA.2.12.1   VVLSFE        516
Omi-BA.4        VVLSFE        516
Omi-BA.5        VVLSFE        516
```

Fig. S9. Sequence alignment of spike RBD from most VOCs used to derive the epitope logo. 002-S21F2 epitope residues are highlighted in green, residue. Residue positions within the 002-S21F2 epitope that are mutated in Omicron are shown as a red arrow.

Table S1: Time points chosen post initial COVID-19 diagnosis for memory B cell characterization

Subject #	Gender	Age	Time post initial SARS-CoV-2 RT-PCR diagnosis (in days)
1	M	35	51
2	M	49	45
3	M	52	56
4	M	44	42
5	M	49	25

Table S2: CryoEM data collection.

**Spike-mAb**	**002-S21F2 WA.1**	**002-S21F2-Omicron**
EMDB	EMD-26262	EMD-26669
Microscope	Talos Arctica	Talos Arctica
Voltage (kV)	200	200
Detector	Gatan K3	Gatan K3
Magnification (nominal)	79000	79000
Energy filter slit width (eV)	20	20
Calibrated pixel size (Å/pix)	1.1	1.1
Exposure rate (e^-/ Å2/sec)	12.75	12.75
Frames per exposure	48	48
Total electron exposure (e^-/Å^2)	51	51
Exposure per frame (e^-/Å^2)	1.0625	1.0625
Defocus range (µm)	1-2.4	1-2.4
Automation software	EPU	EPU
# of Micrographs used	3863	4553
Particles extracted	2,249,333	2,553,301
Particles after 2D classification	457,277	310,886
Total # of refined particles	129,931	108,816
Symmetry imposed	C1	C1
Map sharpening B-factor	148.8	167.6
Unmasked Resolution at 0.5/0.143 FSC (Å)	8.0/4.0	8.7/4.7
Masked resolution at 0.5/0.143 FSC (Å)	4.2/3.8	4.5/4.1

Table S3: **Model refinement and validation statistics**

PDB	7U0P	7UPL
Composition		
Amino acids	**4102**	**4031**
Glycans	**32**	**54**
RMSD bonds (Å)	**0.004**	**0.004**
RMSD angles (º)	**0.605**	**0.65**
Mean B-factors		
Amino acids	**102.42**	**140.31**
Glycans	**135.35**	**189.47**
Ramachandran		
Favored (%)	**96.43**	**93.55**
Allowed (%)	**3.57**	**6.42**
Outliers (%)	**0.0**	**0.0**
Rotamer outliers (%)	**0.39**	**0.0**
Clash score	**5.24**	**7.1**
C-beta outliers (%)	**0.00**	**0.0**
CaBLAM outliers (%)	**2.93**	**3.6**
CC (mask)	**0.71**	**0.74**
CC (volume)	**0.69**	**0.73**
MolProbity score	**1.52**	**1.81**
